# Alginate Self-Crosslinking Ink for 3D Extrusion-Based Cryoprinting and Application for Epirubicin-HCl Delivery on MCF-7 Cells

**DOI:** 10.3390/molecules27030882

**Published:** 2022-01-27

**Authors:** Giulia Remaggi, Ovidio Catanzano, Fabiana Quaglia, Lisa Elviri

**Affiliations:** 1 Department of Food and Drug Science, University of Parma, Parco Area delle Scienze 27/A, 43124 Parma, Italy; giulia.remaggi@unipr.it; 2 Institute for Polymers, Composites and Biomaterials (IPCB-CNR), Via Campi Flegrei 34, 80078 Pozzuoli, NA, Italy; ovidio.catanzano@ipcb.cnr.it; 3 Drug Delivery Laboratory, Department of Pharmacy, University of Napoli Federico II, Via Domenico Montesano 49, 80131 Napoli, Italy; fabiana.quaglia@unina.it

**Keywords:** 3D printing, internal gelation, alginate, biomaterial ink, epirubicin-HCl, MCF-7

## Abstract

3D-printed hydrogels are particularly advantageous as drug-delivery platforms but their loading with water-soluble active compounds remains a challenge requiring the development of innovative inks. Here, we propose a new 3D extrusion-based approach that, by exploiting the internal gelation of the alginate, avoids the post-printing crosslinking process and allows the loading of epirubicin-HCl (EPI). The critical combinations of alginate, calcium carbonate and d-glucono-δ-lactone (GDL) combined with the scaffold production parameters (extrusion time, temperature, and curing time) were evaluated and discussed. The internal gelation in tandem with 3D extrusion allowed the preparation of alginate hydrogels with a complex shape and good handling properties. The dispersion of epirubicin-HCl in the hydrogel matrix confirmed the potential of this self-crosslinking alginate-based ink for the preparation of 3D-printed drug-delivery platforms. Drug release from 3D-printed hydrogels was monitored, and the cytotoxic activity was tested against MCF-7 cells. Finally, the change in the expression pattern of anti-apoptotic, pro-apoptotic, and autophagy protein markers was monitored by liquid-chromatography tandem-mass-spectrometry after exposure of MCF-7 to the EPI-loaded hydrogels.

## 1. Introduction

Three-dimensional (3D) printing is a rapidly growing technology that offers many opportunities for manufacturing biomaterials with tunable properties. The flexibility in the design of this technique allows to fabricate 3D structures with customizable features and intricate architecture and, most importantly, ease of personalization [1]. The extrusion-based approach is probably the most widely utilized method in 3D printing to create structures with a single material via an individual additive manufacturing process [2]. The computer-aided design (CAD) associated with this technology allows to achieve high automation/repeatability and precise control of microstructures, to prepare complex structures in a range of dimensions and material using the layer-by-layer deposition [2,3]. These features are particularly advantageous in the preparation of modified release systems as the conventional drug-delivery platforms often restrict their application in the pharmaceutical industry, due to the incapability of adapting to individual pharmacokinetic traits. Advanced drug-delivery devices with personalized drug dosing and/or complex drug-release profiles, personalized topical treatment devices, and novel dosage forms such as 3D-printed multilayer polypills are just some of the many possible applications of 3D printing in the drug-delivery field [4]. Hydrogels are one of the most feasible classes of ink materials for 3D printing, and this field has been rapidly advancing [5]. A polymer hydrogel is a 3D cross-linked network of flexible polymer chains that contains a large amount of water but retains the properties of solids [6], and they have attracted increasing attention for bioprinting due to the high drug-loading efficiency and excellent biocompatibility [7,8]. Many synthetic and naturally derived hydrogel-forming polymers were proposed as ink material for 3D printing. However, not all the hydrogel materials are suitable for this application, as the printability of a hydrogel is determined by the rheological properties (viscosity, shear-thinning, and yield stress) and cross-linking mechanisms (physical cross-linking and chemical cross-linking) [5]. The interest in natural polymers (often referred to as biopolymers due to their human, animal, plant, and bacteria origins) is due to their excellent biocompatibility and cell affinity, and increasing efforts have been focusing on the use of polysaccharides (sugars linked by *O*-glycosidic bonds), glycosaminoglycans (polysaccharides with amine functionality), and polypeptides/proteins to obtain 3D-printable hydrogels [9]. Alginate (ALG) is a biodegradable, non-toxic, and non-immunogenic natural polymer extracted from marine brown algae biomass, and it is among the most used biopolymers for biomedical applications [10]. ALG is an anionic and hydrophilic block copolymer consisting of ß-d-mannuronic acid (M) and α-L-guluronic acid (G) residues in different proportions, mainly depending on the source. Physical properties and the molecular weight of ALG strictly depend on the sequence of M and G units, as well as the cross-linking which occurs by the co-operative binding of divalent cations (such as Ca^2+^, Ba^2+^, Zn^2+^, or Sr^2+^) with the carboxylic groups of the G-block regions of the copolymer. This interaction results in the formation of a complex interconnected bulk structure in the shape of an “egg-box” [11,12]. The essential role of the crosslinking process in the alginate scaffold design has been extensively reported in the literature [13], but when it comes to 3D printing, this role is even more important. The major drawback of single-component alginate bioink in the form of low-concentration sols (<6 wt%) is the lack of the mechanical strength required to ensure the structural fidelity immediately after extrusion to avoid collapse and deformation. To tackle this problem, He and coworkers proposed to extrude an alginate sols directly into a CaCl_2_ solution to maintain the structural integrity of whole 3D scaffolds during the printing process [14]. Although this method worked for the preparation of high-strength 3D hydrogel constructs with built-in microchannels, its application in drug delivery can be limited by the fast drug release in the crosslinking solution. Moreover, the direct contact with the highly concentrated calcium salt solution needed for the crosslink lead to a rapid and inadequately controlled gelation resulting in hydrogels with poor structural homogeneity and limited mechanical properties [15,16]. Several other strategies were proposed to prepare 3D-printed porous alginate scaffolds, but they all involve contact with calcium salt solutions to ensure the structural integrity of the whole 3D scaffold [17]. Internal gelation is a cross-linking technique that involves the gradual generation of cross-linking ions (Ca^2+^) into the polymeric mixture through a CaCO_3_–GDL (d-glucono-δ-lactone) system. This methodology ensures a slow and more modular gelling process allowing the production of homogeneous hydrogels with well-defined porosity, mechanical properties, and the possibility to include water-soluble actives in the formulation [16,18]. It is clear that a bioink based on this technique could offer several advantages for 3D printing, but, to date, few efforts have been made in this direction. Recently, Hazur and coworkers used internal gelation as a pre-crosslinking technique, to form an homogeneous alginate bioink able to maintain shape fidelity after printing [19]. The authors suggested the feasibility of this method by observing the high cell viability of printed cells embedded in the formulated bioink; however, to fully solidify the 3D-printed structures, a post-crosslinking step was still performed, slowly covering the alginate hydrogels with CaCl_2_. The general aim of this work was to explore the internal gelation technique to prepare an alginate ink capable of completely self-crosslinking after printing. Avoiding the post-printing crosslinking step would make this method more suitable for the preparation of drug-delivery platforms for small hydrophilic molecules. To present a proof of principle, we decided to load our 3D-printed platforms with epirubicin-HCl (EPI), a water-soluble 4′epi-isomer of doxorubicin with a well-established application in breast-cancer treatment, one of the most prevalent diseases among women worldwide [20,21]. EPI induces DNA damage by inhibiting the topoisomerase II activity, inducing apoptosis in cancer cells [22]. EPI local delivery has been extensively studied mainly through the development of nanostructured delivery systems [23,24], but few efforts were made to prepare polymeric platforms for local delivery. One of these attempts proposed EPI-loaded gelatin hydrogel carriers for intravesical usage exhibiting a promising anti-cancer effect on a urothelium carcinoma [25]. This study can be divided into two main parts: the development of an alginate ink based on the combination of ALG/CaCO_3_/GDL to obtain a formulation compatible in terms of viscosity and the gelation time with the extrusion-based 3D. Then, hydrogels functionalized with EPI were printed, characterized, and subsequently tested in vitro on MCF-7 cells, a commonly used human-breast-cancer target cell line [26,27,28]. Finally, using a state-of-the-art liquid-chromatography mass-spectrometry technique, the protein expression of pro- and anti-apoptotic biomarkers was evaluated by target analysis to further validate the potentiality of this approach.

## 2. Results and Discussion

### 2.1. Optimization of the Self-Crosslinking Formulation for 3D Printing

The goal of this work was the development of an ALG-based self-crosslinking bioink to be used in a 3D-printing extrusion-based system. Thus, fine control on the gelation speed is fundamental to obtain homogeneous formulations with such initial viscosities (8–40 kcP) to be printed by the custom low-temperature extrusion 3D-printing system developed for this work [8,29,30,31]. Increasing the viscosity of the initial solution (working at high alginate concentration or preparing blends of polymers) has been a strategy often used to obtain ALG bioinks suitable for 3D printing. However, the high viscosity of the initial solution results in a longer gelation time due to fewer alginate chains mobility and slower diffusion of the crosslinking ions into the bulk material [16]. The internal gelation method, consisting of a combination of a slowly hydrolyzing lactone (GDL) with water-insoluble carbonate salt as a donor of the crosslinking ions, allows instead to generate these ions directly from the inside the material solution, resulting in a hydrogel with suitable viscosity and capable of retaining shape after 3D-printing.

As a first step, we tried to establish the best combinations of GDL concentration and scaffold production parameters that would allow us to obtain self-crosslinked 3D hydrogels. The concentration of GDL in the bioink formulation is the critical parameter to modify gelation properties of ALG [16], as too-fast gelation can lead to difficulties in printing the material, while too-slow gelation results in low-extrusion printing precision and large variation in the device shape after printing. Our strategy to avoid the use of a too-high concentration of GDL (that would have started the gelation in the syringe used for the extrusion of the alginate) was to use a low temperature to maintain the 3D structure after printing, making the extrusion take place on a Peltier cell at −14 °C. However, it should be noted that the ink preparation and the printing process occur at room temperature, whereas only the post-printing cross-linking reaction was performed at low temperature (2 °C). As the temperature significantly affects the kinetic of the ALG GDL-mediated self-crosslinking reaction, and thus the ink viscosity over time, the optimization of the whole scaffold production process required the study of the effect of the molar ratio between CaCO_3_ and GDL on the ALG gelation properties. As a starting point, we took into consideration some previous studies that have shown that is possible to prepare self-crosslinking alginate hydrogels scaffolds with defined dimensions for tissue-engineering applications with a molar ratio between CaCO_3_ and GDL of 0.5 [15] and 2, in the case of alginate–hyaluronan blends [16].

As a preliminary test, a solution of ALG (6% *w*/*v*) and CaCO_3_ (80 mM) was mixed, in a 1:1 *v/v* ratio, with GDL 80 mM and loaded into a syringe to be printed. A four-layer scaffold was successfully printed on the plate of Peltier cell (−14 °C) and left to crosslink first for 10 min at −20 °C to avoid 3D structure collapse and then at 2 °C. The scaffold was monitored over time, and only after 5 days it easily detached from the plate and was able to retain its 3D structure at room temperature. However, once immersed in water, it dissolved immediately, clearly indicating that a complete dehydration process of the formulation occurred instead of a cross-linking reaction.

Starting from this evidence, we decided to fix the initial concentration of ALG and CaCO_3_ on 6% (*w*/*v*) and 160 mM, respectively. Once the experimental conditions for the formation of the bioink were fixed, the influence of GDL on the printing time was evaluated. For this purpose, GDL solutions at increasing concentrations (from 120 mM to 180 mM) were prepared and mixed with the ALG/CaCO_3_. A series of technological parameters such as the ink working time, the number of printable layers, the scaffold curing time at 2°C, the scaffold detachment from the plate, and the retention of the 3D structure in water were evaluated. Table 1 summarizes the main findings for the different combinations of ALG/CaCO_3_/GDL tested.

**Figure 1:**
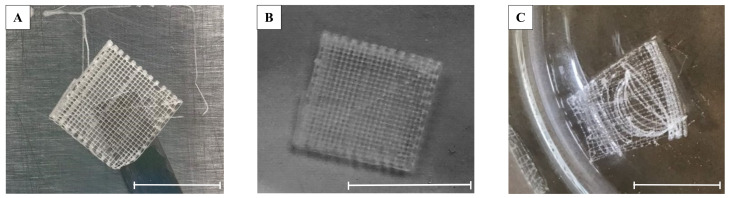
Alginate-based 3D-printed scaffold. (**A**) 3-layers-160 mM scaffold detached correctly from the plate; (**B**) 4-layers-140 mM self-crosslinked scaffold in water retained the 3D structure; (**C**) 6-layers-120 mM scaffold in water flaked off; the crosslinking reaction was not successful. Scale bar: 1 cm.

The formulation with the highest GDL concentration (180 mM) was immediately discarded, as the too-fast gelation (ink working time: 8 min) allowed to print hydrogels with only two overlapping layers. The formulation crosslinked with 160 mM GDL presented a longer printing time of approx. 11 min, enough to obtain a three-layer scaffold able to be easily detached from the plate (Figure 1a) and to maintain the 3D structure if left for 2 to 4 days at 2 °C. Similarly, the formulation with 140 mM GDL showed a good printability with an extended printing time with the possibility of preparing hydrogels with four overlapped layers and a similar self-gelling capacity (Figure 1b). The poor crosslinking ability confirmed by a difficult detachment from the plate and the fast dissolution of the hydrogel 3D structure when placed in water forced us to discard the 120 mM GDL formulation (Figure 1c).

The data reported on Table 1 evidenced a linear relationship (Y = −0.165X + 37.5) between the GDL molar concentration (X) and the ink working time (Y) that, together with the experimental evidence here collected, can be useful to predict the behavior of the formulation over the processing time.

The alginate-based 3D-printed scaffold formed by 6% *w/v* ALG, CaCO_3_ 160 mM and 140 mM GDL crosslinked for 3 days at 2 °C (3D-ALG) resulted to have the best overall characteristics, and for this reason, they were chosen for further characterization and EPI loading. In particular, we decided to set the curing time of 3 days at 2 °C to ensure the maximum crosslinking rate, avoid hydrogel dehydration during the crosslink time, and retain the 3D-printed structure. EPI-loaded hydrogels were prepared to the final concentrations of 0.6 and 1 µg per hydrogel (named ALG_EPI_0.6 and ALG_EPI_1, respectively) as described in the experimental section. As no differences in scaffold production were experimentally evidenced after drug addition, we conclude that EPI did not affect the processing parameters described above.

### 2.2. ATR FT-IR Scaffold Characterization

**Figure 2:**
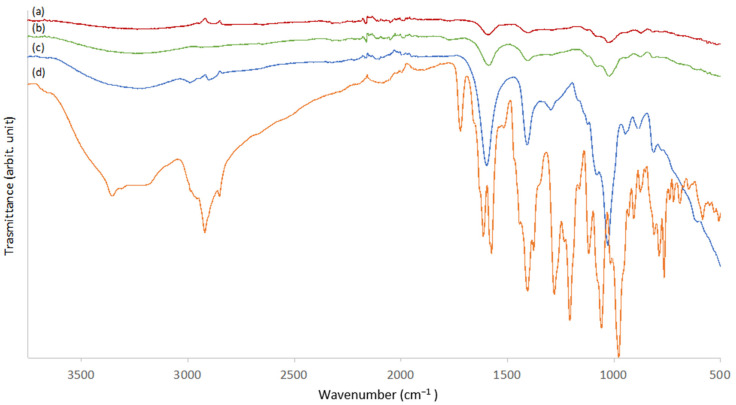
ATR FT-IR spectra of the 3D-printed samples. (a) 3D-ALG, (b) ALG_EPI_1, (c) ALG, (d) EPI.

The ATR FT-IR spectrum of raw alginate powder was compared with the spectra of self-crosslinked 3D-printed hydrogel (3D-ALG) in order to chemically characterize the new formulation developed (Figure 2c). In the alginate powder spectrum, the wide peak between 3000 and 3600 cm^−1^ corresponds to the −OH stretching vibration. Stretching vibrations of aliphatic C–H were observed at 2986–2898 cm^−1^. Bands at 1601 and 1412 cm^−1^ can be attributed to asymmetric and symmetric COO− stretching vibrations of carboxylate salt groups. The bands at 1032 and 947 cm^−1^ were attributed to the C–O stretching vibration of a pyranosyl ring and the C–O stretching with contributions from C–C–H and C–O–H deformation. Finally, at 886 cm^−1^ the vibration of a mannuronic acid functional group was shown [27,32]. Comparing this spectrum with 3D-ALG, the latter presents general bands broadening and peak shifts. In 3D-ALG (Figure 2a), the asymmetric stretching vibration of carboxylate ion shifted to significantly lower wave numbers (i.e., from 1601 to 1586 and from 1412 to 1404 cm^−1^), whereas the vibration of the mannuronic-acid functional group shifted from 886 cm^−1^ to 874 cm^−1^. This behavior can be attributable to the presence of calcium ions and to their cross-linking effects involving the carboxylate groups that are diagnostics of the changes in the structure of alginate. The ATR FT-IR spectrum of a similar alginate hydrogel obtained by immersion in a CaCl_2_ solution was even acquired as a reference to compare the alginate intermolecular structures obtained after 3D printing. Both the spectra obtained were superimposable, and no significant differences in the vibration wave numbers were observed (data not shown). This finding suggests that the CaCO_3_/GDL cross-linking system produced a final alginate structure very similar to that obtained by conventional cross-linking in a free Ca^2+^ ion solution. Figure 2b shows the spectrum of ALG_EPI_1 hydrogel. No significant differences between the spectra of ALG and ALG_EPI_1 were observed in terms of peak wave numbers. However, in the ALG_EPI_1 spectrum, the bands at 1587 cm^−1^ and 1404 cm^−1^ were more intense compared to the control scaffold. The EPI ATR FT-IR spectrum (Figure 2d) exhibited two intense bands at 1574 cm^−1^ and 1404 cm^−1^ that overlap with the bands of alginate scaffold. Even the band at 1030 cm^−1^ was more intense in the ALG_EPI spectrum compared to the ALG spectrum. On the contrary, the vibration of the mannuronic-acid functional group at 874 cm^−1^ resulted in more intensity in the 3D-ALG. These findings suggest the presence of EPI in the scaffold and its effect of the ATR FT-IR spectrum even if the drug was present at the amount of 1 μg/scaffold.

### 2.3. Swelling Behavior and Drug Release from 3D-Printed Hydrogel

**Figure 3:**
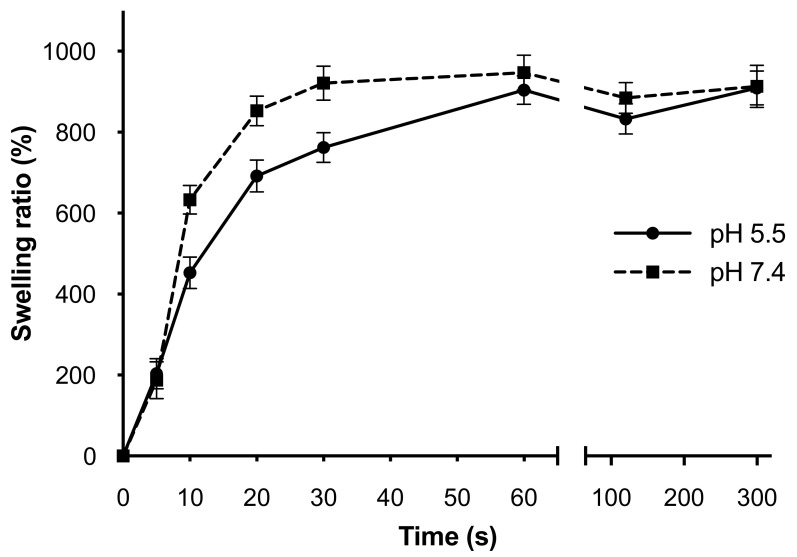
Swelling profile of the 3D-ALG at different pH. Swelling profiles at pH 7.4 (dotted line) and pH 5.5 (continuous line) were shown; results are reported as mean ± standard deviation of three independent measurements.

The release of drugs from hydrophilic crosslinked networks strongly depends on the swelling behavior. The ability of a hydrogel to absorb and retain biological fluids is strictly related to the physical structure and the characteristics of the fluid used to simulate the physiological condition of use. The presence of a crosslinked network generally modifies the swelling of a hydrogel, reducing the swelling ratio as a consequence of a more rigid structure. The rate and duration of swelling for 3D-ALG were studied over a five-minute period using sodium acetate buffer (pH 5.5) or PBS solution (pH 7.4) at 37 °C to verify if the slightly alkaline or acidic physiological condition may affect the swelling behavior. Figure 3 shows a similar trend in both pH conditions, with a rapid initial increase in water uptake in the first 60 s of the experiment followed by an equilibrium state until the end. In the first minute after the immersion, the swelling kinetics were faster for the hydrogel placed at pH 7.4, then the behavior at the two pH levels became more and more uniform, overlapping between 60 and 300 s and stabilizing on a rehydration percentage of about 900%.

In the further step, the release of EPI from the 3D-printed hydrogel was followed to evaluate the ability of the system to release an hydrophilic molecule. The EPI actual amount in the hydrogel was experimentally quantified by using the HPLC-UV/VIS method specifically developed, resulting in an actual loading of 72.3 ± 7.5%. This value, evaluating the process production in its totality, can be considered acceptable; thus, the process developed to obtain a self-crosslinking alginate 3D-printed hydrogel allowed to successfully functionalize scaffolds with water-soluble drugs such as EPI.

**Figure 4:**
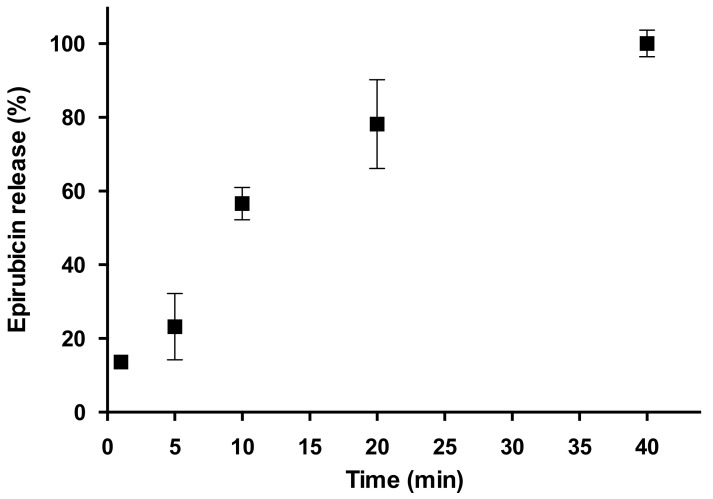
EPI-release profile from ALG_EPI _1 in PBS pH 7.4. Results are reported as mean ± standard deviation of three independent measurements.

The release profile of EPI from the ALG_EPI_1 scaffold in PBS at pH 7.4 and 37 °C is reported in Figure 4. Usually, there is a direct correlation between the drug release from biodegradable polymeric matrices and the water-uptake kinetics (i.e., swelling), as the force that drives the drug release of hydrophilic molecules is the entrance of water into the matrix [18,33]. In this case, the drug-release experiment showed a slow release of EPI up to 40 min, a much longer time compared with the 5 min required to reach the maximum hydration state of the hydrogel. This difference could be explained if we also consider the internal structure of the hydrogel. A crosslinked polymeric network consists of a series of open spaces (meshes) between polymer chains that allow for liquid and small-solute diffusion. The mesh size depends on the polymer and crosslinker concentrations, as well as external stimuli such as temperature and pH [34]. In our case, we reasonably believe that the 40 min needed to reach 100% of the drug release are due to the complex internal structure/interactions of the ALG_EPI_1 scaffold that slow down the diffusion of active molecules. Finally, it is worth to notice that no drug-degradation phenomena were detected under pH and temperature conditions used in accordance with the existing literature on EPI [35].

### 2.4. Cytotoxic Activity towards MCF-7 Breast-Cancer Cells

**Figure 5:**
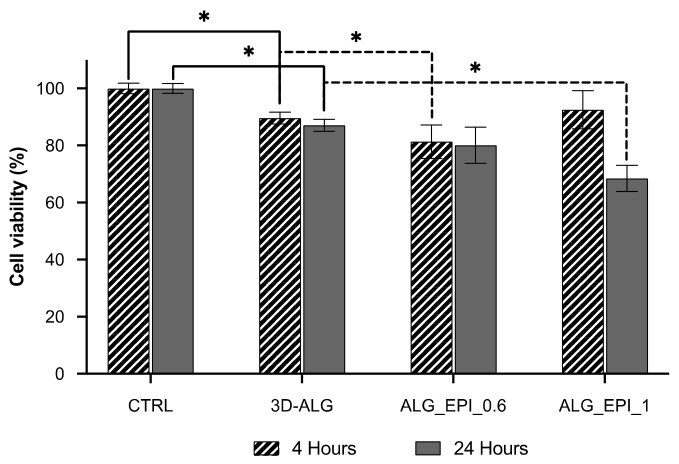
Effect of self-crosslinking 3D-printed hydrogel on MCF-7 cells viability. Cell viability was determined by resazurin assay at 4 h (diagonal lines) and 24 h (grey fill) on the nude plate (CTRL), 3D-ALG, and hydrogels containing 0.6 (ALG_EPI_0.6) and 1 g (ALG_EPI_1) of the drug, respectively. Results are reported as mean ± standard deviation of three independent measurements. Statistically significant values were denoted by * (*p* < 0.05; One-way ANOVA test).

The cytotoxic activity of both unloaded ALG and loaded ALG_EPI_1 hydrogel was tested on MCF-7 cells using resazurin assay. MCF-7 cells are often used as in vitro breast-cancer models, and they proved to be particularly sensitive to the action of EPI [36]. According to the recommended guidelines for the evaluation of in vitro cytotoxicity for medical devices and delivery systems (ISO 10993-5:2009), a biomaterial can be deemed non-cytotoxic if the cell viability after exposure does not fall below 70% [37]. The biocompatibility test carried out at both 4 h and 24 h and reported in Figure 5 showed a cell viability significantly lower if compared to the control on the nude plate (CTRL). However, these values (87–89%) fall within the biocompatibility threshold defined by the recommended guidelines, allowing us to consider the delivery platform as biocompatible. Once loaded with EPI, the cytotoxic behavior changed significantly. After just 4 h of exposition to the drug-loaded scaffold, even ALG_EPI_0.6 (with the lowest drug amount, 0.6 µg) significantly reduced cell viability compared to the CTRL. However, as expected, ALG_EPI_1 (loaded with 1 µg of EPI) gave the best results in terms of cytotoxicity, with a reduction in the cell viability by about 19% compared to CTRL after 24 h. These observations suggest the efficacy of the developed 3D-printed hydrogel to perform cytotoxic effects on MCF-7 cells with an effect directly dependent on the drug concentration.

### 2.5. Proteomic Analysis

**Figure 6:**
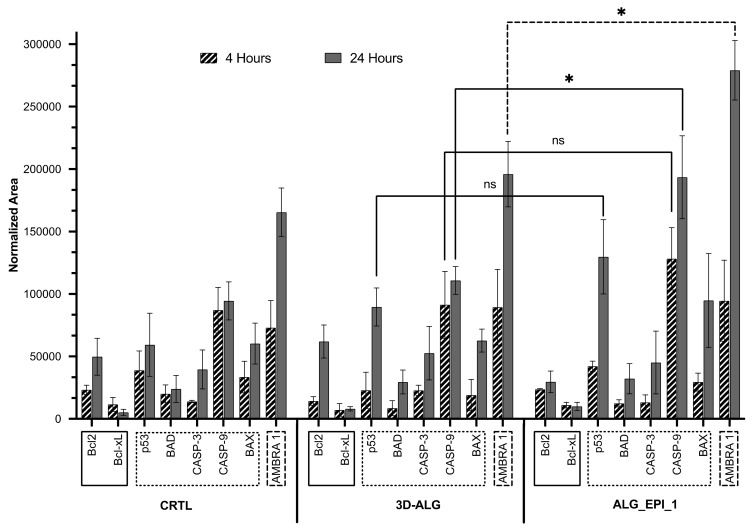
LC-MS/MS SRM-targeted protein analysis. Proteins’ expression at 4 h4h (diagonal lines) and 24 h (grey fill) for CTRL, 3D-ALG, and ALG_EPI_1 (1 µg drug loaded) treatment conditions were shown. Proteins are grouped according to their biological function: anti-apoptotic (continuous line), pro-apoptotic (dotted line), and involved in autophagy (dashed line). Results are reported as mean ± standard deviation of five total replicates (three biological replicates and two instrumental replicates); statistically significant values were denoted by (* *p* < 0.05; one-way ANOVA test), whereas not-significant ones were denoted as ns (*p* > 0.05; one-way ANOVA test).

In an endeavor to further investigate the effect of ALG_EPI_1 on the MCF-7 cell line, the main proteins involved in the processes of apoptosis were analyzed by LC-MS/MS SRM target analysis. In particular, we studied the anti-apoptotic Bcl-2 and Bcl-xL class proteins; the pro-apoptotic p53, BAX, and BAD proteins; the executioner caspase 3 (CASP-3); the initiation caspase 9 (CASP-9); and also the activating molecule in BECN1-regulated autophagy protein 1 (AMBRA1), which are all proteins involved in the MCF-7 autophagy program as a mechanism of protection from cell death induced by EPI [38]. The presence/absence of such proteins and their relative quantification was performed after 4 h and 24 h of exposition both on the ALG and ALG_EPI_1 using the cells cultured on the nude plate as the control group (CTRL). The results of this analysis are summarized in Figure 6.

Bcl-2 and Bcl-xL are two proteins involved in the anti-apoptotic process. BCl-xL was expressed at similar levels in all samples. It worth to notice that the Bcl-2 expression rose significantly (*p* < 0,05) from 4 to 24 h in the CTRL and 3D-ALG hydrogel, confirming the biocompatibility of these two systems. Whereas in the ALG-EPI_1, no increase was observed over this time range.

Regarding pro-apoptotic proteins, their expression varies over time and between treatments. The expression of the p53 protein increased over time and was more expressed in the ALG_EPI_1 samples with respect to CTRL and 3D-ALG, suggesting that the loaded drug started to induce DNA damage [39]. BAD, CASP-3, and BAX protein expressions were comparable between the hydrogel tested, increasing over time. Interestingly, the CASP-9 expression level was similar between the CTRL and 3D-ALG and significantly increased in the drug-treated samples at 24 h. CASP-9 is an initiating caspase upstream of CASP-3 [40], unlike CASP-3, and differs significantly in expression from the 3D-ALG (*p* < 0.05), strengthening the thesis of ALG_EPI-mediated apoptosis induction that is only in the early phase.

Finally, AMBRA1 expression, a marker of the autophagy cell process, increased over time in all samples. CRTL and 3D-ALG treatments showed a quite similar effect on MCF-7 cells at both times, whereas at 24 h, AMBRA1 expression significantly increased (*p* < 0.05) in ALG_EPI_1, confirming the cytotoxic effect of the hydrogel also evidenced by the resazurin assay. MCF-7 cancer cells, if treated with drugs that induce cell death such as EPI, can trigger the autophagy process as a defense mechanism [20]. These outcomes confirm some of those obtained from the viability assays and reveal that ALG_EPI_1 could be used as drug-delivery systems to induce target cells in the early stage of apoptosis.

## 3. Materials and Methods

### 3.1. Reagents

Sodium alginate (Ph.Eur. grade; molecular weight by gel-filtration chromatography (GFC) 180–300 kDa; slowly soluble in water) was from Carlo Erba (Milan, Italy). Urea, tribasic sodium citrate dihydrate, and calcium carbonate (CaCO_3_) were from Carlo Erba (Milan, Italy). Trypsin from bovine pancreas, bovine serum albumin (BSA), glucono-δ-lactone (GDL), ethylene diamino tetraacetic acid (EDTA), DL-ditiothreitol (DTT), iodoacetamide (IAA), ammonium bicarbonate (ABC), sodium acetate, formic acid (FA), and acetonitrile (ACN) were from Sigma-Aldrich^®^ (Darmstadt, Germany). Dulbecco’s Modified Eagle’s Medium (DMEM), Steptomycin, and Penicillin was from Gibco (Thermo Fisher Scientific, Waltham, MA, USA). Fetal Serum Bovine (FBS) and Dulbecco’s phosphate buffered saline (PBS) were from Euroclone (Milan, Italy). Epirubicin-hydrochloride (EPI) was obtained from Cayman Chemical (Ann Arbor, MI, USA). Protein assay dye reagent concentrate was from Bio-Rad. Resazurin sodium salt was from Alfa Aesar (Ward Hill, MA, USA). All solvents were of reagent grade with the highest purity available. Deionized ultra-filtered water was used throughout this study.

### 3.2. Ink Preparation for 3D Printing

Sodium alginate (ALG) (6% *w*/*v*) and calcium carbonate (CaCO_3_) (80 and 160 mM) powders were dispersed in ultrapure water in a beaker and magnetically stirred for 48 h until a homogeneous dispersion was achieved. One mL of the ALG/CaCO_3_ dispersion was first sonicated in an ultrasonic bath (Branson 2510; OPTO-LAB, Modena, Italy) for 30 min at room temperature and then carefully and quickly mixed with 1 mL of a freshly prepared GDL water solution. Different GDL concentrations (120, 140, 160 and 180 mM: pH= 4.5 ± 1) were tested. It is worth to notice that once the two solutions were mixed, the final concentration of ALG was 3%, and the concentrations of CaCO_3_ and GDL were halved. For drug-loaded hydrogels, an EPI water solution was added drop by drop into ALG/CaCO_3_ dispersion to achieve a final concentration of 6 μg/mL and 10 μg/mL.

### 3.3. Scaffold Design

Three-dimensional models of the hydrogels were drawn using the Computer-Aided Design (CAD) software as previously reported [31,41]. Briefly, solidworks TM (Dassault systems, Waltham, MA, USA) was used for this purpose, allowing the creation of a “.stl” file (STereo Lithography interface format). This file was further processed by Slic3r (RepRap), a slicing program that generates the machine code (GCode) file for the 3D printer. The three-dimensional models were built by overlapping two figures presenting parallel strands and rotating them by 90°. In total, 1 to 4 layers were printed, forming an orthogonal grid with fixed distances between filaments of 200 μm and creating a three-dimensional “gauze” shaped like a net of 1.6 cm × 1.6 cm in size.

### 3.4. 3D Printer and Scaffold Manufacturing

A custom-built low-temperature manufacturing system was designed and developed by combining Peltier cells and liquid/air exchangers with a commercial Direct Ink Writing (DIW) 3D printer previously described [31,41]. The ink formulation formed by ALG, CaCO_3_, and GDL was loaded into a 5 mL syringe and extruded through a 26 G transversely cut needle (inner diameter 0.260 mm) by the application of controlled pressure on the syringe through a mechanically assisted piston. The deposition took place through a robotic arm that moves along three axes (x, y, z) following the directives imposed by the GCode file on a stainless-steel Peltier cell (−14 °C). For each ink formulation, a 4-layer square hydrogel was printed in triplicate using approximately 100 μL of the solution, resulting in a scaffold loaded with 0.6 µg or 1 µg of EPI. Once the deposition was completed, the scaffold was further frozen at −20 °C for 10 min and then left at 2 °C for a time that range from 1 to 5 days to complete the self-gelation process. After the gelation time, the hydrogels were brought to RT and detached from the steel plate. Once detached from the plates, the hydrogels obtained were stored at 4 °C until use.

### 3.5. Swelling Test

The hydrogels’ swelling behavior was evaluated by measuring the fluid uptake as a function of time. After printing and crosslinking (3 days at 2 °C), the 3D-ALG hydrogels were dried in vacuum oven at 30 °C for 24 h, and the initial weight of each sample was accurately recorded using an analytical scale. The fluid uptake was evaluated by placing the samples in sodium acetate buffer (20 mM; pH 5.5) on in Phosphate Buffer Saline solution (PBS, NaCl 120 mM, KCl 2.7 mM, Na2HPO4 10 mM, pH 7.4) in a thermostatic bath at 37 °C. At specific times (5, 10, 20, 30, 60, 120, and 300 s) the samples were taken out, excess fluid was carefully removed using tissue paper, and after being weighed were re-immersed in the solutions. The percentage swelling ratio (SR%) at each time point was calculated using Equation (1):

SR% = [(W − W_0_)/W_0_] × 100 (1)

where W is the mass of the swollen sample, and W_0_ is the mass of the initial dry sample.

### 3.6. Attenuated Total Reflection (ATR) Fourier Transform Infrared (FT-IR) Spectroscopy

ATR FT-IR spectra were obtained using a Perkin–Elmer spectrometer (Norwalk, CT, USA). The apparatus operates with a single reflection at an incident angle of 45°. The analysis was carried out on hydrogels and raw materials at room temperature and ambient humidity. For each spectrum, 32 scans were acquired between 4000 and 400 cm^−1^ with a spectral resolution of 2 cm^−1^.

### 3.7. Evaluation of the Epirubicin-HCl Loading

The EPI actual loading in hydrogels was evaluated by dissolving a single 4-layers scaffold, previously dried and weighted to calculate the theoretical EPI content from ink composition, in 400 μL of a solution of sodium citrate (55 mM) and EDTA (50 mM) at pH 7.4 under magnetic starring. The EPI loaded content (EPI%) was evaluated by liquid chromatography-ultraviolet/visible spectrophotometry (HPLC-UV/VIS) using Equation (2)

EPI% = M/M_0_ × 100 (2)

where M is the amount of EPI experimentally determined, and M_0_ is the amount of EPI calculated theoretically from the composition % of the initial dried scaffolds.

EPI analysis was carried out by RP-HPLC using an LC Agilent 1200 system equipped with a UV/VIS detector (Agilent Technologies, Santa Clara, CA, USA). The analysis was performed at 25 °C on a Luna C18 column (50 mm × 2.1 mm, 5 µm particles) (Phenomenex, CA, USA). The mobile phase consisted of aqueous formic acid 0.1% solution (*v*/*v*) and acetonitrile at the ratio 50:50. The elution was isocratic at a flow rate of 200 μL/min; the detector wavelength was fixed at 479 nm; and the injection volume was 10 μL in all the experiments. Each sample tested was injected in duplicate.

### 3.8. In Vitro Drug Release Studies

The in vitro release profile of EPI from ALG_EPI_1 was evaluated in PBS at pH 7.4. Three hydrogels were first weighed and then incubated in 1.4 mL of PBS and placed in a thermostatic bath at 37 °C. At scheduled time intervals up to 24 h, 30 μL of the release medium was withdrawn and replaced with the same volume of fresh medium. The collected samples were analyzed for EPI content by high-performance liquid-chromatography tandem mass spectrometry (LC-MS/MS) using an LC Agilent HP 1260 (Agilent Technologies, Santa Clara, CA, USA) equipped with a 200-vial capacity sample tray and coupled to a QTRAP 4000 triple quadrupole mass spectrometer (ABSCIEX, CA, USA), interfaced with a pneumatically assisted electrospray source (ESI). A C18-reversed phase column (Atlantis dC18, 2.1 × 100 mm, 100 Å, 3 µm) (Waters, Milford, MA, USA) kept at a temperature of 25 °C was used for chromatographic separation. The mobile phase consisted of an aqueous formic acid 0.1% solution (*v*/*v*) and acetonitrile at the ratio 50:50. The elution was isocratic at a flow rate of 200 μL/min. For the analysis, 10 μL of each sample was injected in duplicate. The sheath gas (nitrogen, 99.999% purity) and the auxiliary gas (nitrogen, 99.998% purity) were delivered at flow rates of 45 and 5 arbitrary unit, respectively. Optimized conditions of the interface were as follows: ESI voltage 5.5 kV, declustering potential (DP) 80 eV, and capillary temperature 350 °C. MS experiments were performed under selective ion monitoring (SIM) conditions. The EPI SRM transition monitored was 566/545 *m*/*z*.

### 3.9. MCF7 Cells Culture

Biological investigation on ALG_EPI_1 was performed on the MCF-7 breast-cancer cell line. MCF-7 cells were cultured at 37 °C with Dulbecco’s modified Eagle’s medium (DMEM) supplemented with 10% FBS, 100 IU/mL penicillin, and 100 IU/mL streptomycin. Cultures were maintained in humidified atmosphere of 95% air and 5% CO_2_ at 37 °C. To test the 3D-printed hydrogels, MCF-7 were seeded in 12-well plates at a density of 5.5 × 10^5^ cells/well and maintained in 2 mL of cell culture media. After 48 h, the medium was changed with a serum-free one, and a scaffold was placed in each well for the time of the experiment.

### 3.10. Cytotoxic Activity towards MCF-7 Breast-Cancer Cells

The cytotoxicity of the self-crosslinked hydrogels was assessed after 4 h and 24 h using the resazurin assay. ALG_EPI_1 and control hydrogels (3D-ALG) were sterilized under UV light for 30 min each side and directly placed in each well. Three replicates were performed for each grown condition (nude plate-CTRL, 3D-ALG, and ALG_EPI_1). At each time point (4 and 24 h), the hydrogels were removed, and the cell media was aspirated; cells were gently washed in PBS to remove eventual debris, and 1.2 mL resazurin solution (10 μg/mL) was added to each well. Plates were incubated for 2 h at dark at 37 °C in a wet atmosphere with 5% CO_2_ and 95% air. Fluorescence was recorded at 540 nm excitation and 590 nm emission by a Spark^®^ microplate reader (Tecan “Spark 10M”; Software Sparkcontrol Method Editor Tecan, Switzerland). The fluorescence values of the control plate were considered as 100% of viability.

### 3.11. Proteomic Analysis

MCF-7 seeded in 12-well plates were cultured with the 3D-ALG or ALG_EPI_1 for 4 h and 24 h in triplicate. After this time, the hydrogels were gently removed, and the cell culture mediums were taken from each culture well and stored for further analysis. Adherent cells were washed with PBS, detached using trypsin solution (150 μL/well), and collected in a small tube after centrifugation. Cell pellets were further washed with fresh PBS repeated 3 times and stored at −80 °C until use. Total proteins were extracted from cell pellets using an 8 M urea lysis buffer in 50 mM ammonium bicarbonate (pH 8). Lysates were vortexed and put on a stirring plate for 15 min then sonicated for 30 min at 4 °C. Extracts were centrifuged at 4 °C for 10 min at 15,000 rpm, and the supernatants thus obtained were concentrated with buffer exchange procedure using 50 mM ammonium bicarbonate in Amicon Ultra 0.5 mL centrifugal filters with a 3-kDa Molecular weight cut-off. The ensuing protein extracts were assayed for total protein content using the Bradford method. Briefly, 10 µL of each protein extract were added to 500 µL of a diluted Bradford reagent solution (1:5 with water) and incubated for 5 min in the dark at room temperature. After incubation, the ABS of the samples were recorded at a wavelength of 595 nm. The calibration curve was prepared using a BSA standard (1 mg/mL) diluted to a range of 10–400 μg/mL (y = 0.001x; R^2^ = 0.995). The extract was further processed, and the proteins were reduced and alkylated with DTT (100 mM for 30 min at 37 °C), followed by IAA (200 mM for 30 min in the dark), and the addition of DTT (100 mM for 15 min at room temperature). Finally, samples were digested by incubation with trypsin (1:50 enzyme/substrate ratio) at 37 °C overnight and, the digestion stopped with 1 μL of FA added to each sample. All samples were dried under nitrogen flow and stored at −20 °C until use. The tryptic peptides were reconstituted in 100 μL of 0.1% (*v*/*v*) formic acid aqueous solution and 50% (*v*/*v*) acetonitrile before being analyzed with LC-MS/MS to identify and relative quantify pro and anti-apoptotic protein biomarkers using the selected reaction monitoring (SRM) approach. Samples were analyzed as biological (*n* = 3) and technical replicates (*n* = 2) using the same instrumentation reported in the previous section. An Xbridge Peptide BEH C18 (250 × 2.1 mm, 5 μm) column (Waters, Milford, MA, USA) equipped with a pre-filtering column was used for peptide separation. The injection volume was 15 μL. An elution system based on a solvent gradient (solution A: 0.1% aqueous formic acid (*v*/*v*); solution B: acetonitrile) was delivered at 0.2 mL/min. The gradient was set as follows: 0 min 2% solvent B, 4 min 2% solvent B, 150 min 90% solvent B, 155 min 90% solvent B, and 170 min 2% solvent B. As for semiquantitative analysis, experiments were performed under positive ion-SRM conditions using nitrogen as collision gas (pressure of 2.1 × 10^−3^ mbar in the collision cell; collision energy 40 V) and a 20 ms-dual time for each transition monitored. The SRM transitions monitored were reported in Table S1 (Supplementary Materials). The analytes were relatively quantified among samples by normalization with total protein content obtained by Bradford assay.

### 3.12. Bioinformatic Analysis and Mass Spectrometry Data Processing

A panel of eight anti and pro-apoptotic proteins (Bcl-2; Bcl-xL; p53; BAD; CASP-3; CASP-9; BAX; AMBRA1) were considered for the assay. FASTA protein sequences were obtained from Uniprot Human proteome database and SRM transitions for each protein were simulated by Skyline (v. 20.0, SCIEX) (Redwood City, CA, USA), setting trypsin as digestion mode with no missed cleavage and carbamidomethylation of cysteins as structural modification (Table S1). The uniqueness of candidate peptide sequences was assessed by BLASTp tool (basic local alignment search tool; www.ncbi.nml.nih.gov (accessed on 25 December 2021) link NCBI BLAST) search (algorithm: blastp; ATRIX PA 30; GAP COASTS: existence 10, extension 1; DATABASE: non redundant protein sequences) from NCBI (National Center for Biotechnology Information) (Bethesda, MA, USA). LC-MS/MS data were analyzed using the Analyst v 1.4 software, and integrations of the peaks areas were obtained by the MultiQuant program (version 2.1, ABSCIEX).

### 3.13. Statistical Analysis

Statistical analyses were undertaken using GraphPad Prism^®^, version 6.00 (GraphPad Software, La Jolla California USA) using one- way ANOVA test. All experiments were performed in triplicate, and the results were expressed as the mean ± standard deviation (SD). A value of *p* < 0.05 was considered statistically significant.

## 4. Conclusions

A new alginate-based self-crosslinking formulation for 3D printing was successfully developed. Hydrogels were produced by an extrusion 3D printer with a one-step procedure that, by starting the crosslink during the extrusion, avoids the post-printing immersion in a crosslinking bath. The loading test carried out using a model water-soluble drug as EPI confirmed the potential of this procedure for the preparation of 3D-printed hydrogels intended as drug delivery systems. The 3D-printed hydrogels have good handling properties and a swelling behavior typical of hydrogels independently from the pH of the testing solution. The production procedure adopted allows an easy EPI loading, allowing to prepare hydrogels with an accurate drug title and a fast release up to 40 min. In vitro tests on MCF-7 breast cancer cells confirm the biocompatibility of the 3D-ALG hydrogel, with an increase in the cytotoxic effects at 24 h if functionalized with 1 µg of EPI. The cytotoxic potential against MCF-7 was validated by the expression by cells of some apoptosis marker proteins studied in a target MS analysis. Hence, the use of internal gelation can be a promising approach in 3D printing of alginate, allowing the preparation of new functionalized biomaterial for medical applications.

## Figures and Tables

**Table 1:** Alginate-based formulations tested for 3D printing at various concentrations of GDL.

(GDL) mM	Ink Working Time * (Min)	Number of Layers	Curing at 2 °C	Detachment from Plate	Self Cross-Linking
180	8	2	1	no	yes
160	11	3	1	no	yes
			2; 3; 4	yes	
			5	difficult	
140	14	4	1	no	no
			2	no	yes
			3	yes	yes
			4	yes	yes
			5	difficult	yes
120	18	6	1	no	no
			2	no	
			3	yes	
			4; 5	yes	

* Ink working time is intended as the time occurring from the preparation of the ALG/CaCO_3_/GDL mixture to the printable end point.

## Data Availability

The data that support the findings of this study are available from the corresponding author upon reasonable request.

## Supplementary Materials

The following supporting information can be downloaded as following, Table S1: SRM transitions with their *m/z* values selected for LC-MS/MS analysis.

## Sample Availability

Samples of the compounds are available from the authors upon reasonable request.
