# Supplementary material for: Alginate Self-Crosslinking Ink for 3D Extrusion-Based Cryoprinting and Application for Epirubicin-HCl Delivery on MCF-7 Cells
Source: Molecules. 2022 Jan 27;27(3):882. doi: 10.3390/molecules27030882 (PMC8839018; doi:10.3390/molecules27030882)
Supplement: Supplementary file 1 [file molecules-27-00882-s001.zip › molecules-1550773-supplementary.pdf]

Supplementary materials

Alginate self-crosslinking ink for 3D extrusion-based cryoprinting and application for epirubicin-HCl delivery on MCF-7 cells

*Giulia Remaggi, Ovidio Catanzano, Fabiana Quaglia, Lisa Elviri**

*Correspondence: lisa.elviri@unipr.it

Table S1. SRM transitions with their *m/z* values selected for LC-MS/MS analysis. Sequences chosen for semiquantitative evaluation are in bold.

PROTEIN ID	PROTEIN NAME	PEPTIDE SEQUENCE	PRECURSOR (*m/z*)	PRODUCT (*m/z*)
P10415	Apoptosis regulator Bcl-2 (**Bcl-2**)	DFAEMSSQLHLTPFTAR	975,973	1009,430
		**EMSPLVDNIALWMTEYLNR**	**1148,061**	**1369,682**
Q07817	Bcl-2-like protein 1 (**Bcl-xL**)	EMQVLVSR	481,260	488,217
		**WFLTGMTVAGVVLLGSLFSR**	**1077,593**	**1218,720**
P04637	Cellular tumor antigen p53 (**p53**)	**C[+57]SDSDGLAPPQHLIR**	**833,404**	**931,547**
		KPLDGEYFTLQIR	790,428	803,393
		QSQHMTEVVR	607,801	871,445
Q92934	Bcl2-associated agonist of cell death (**BAD**)	MSDEFVDSFK	602,763	742,377
		**SAPPNLWAAQR**	**605,820**	**631,331**
P42574	Caspase-3 (**CASP-3**)	**DGSWFIQSLC[+57]AMLK**	**828,400**	**950,480**
		VATEFESFSFDATFHAK	967,452	998,447
P55211	Caspase-9 (**CASP-9**)	QLIIDLETR	550,819	583,345
		FSSLHFMVEVK	662,342	850,392
		**LSKPTLENLTPVVLRPEIR**	**1088,147**	**1097,620**
Q07812	Apoptosis regulator BAX (**BAX**)	**VVALFYFASK**	**572,824**	**615,314**
		MGGEAPELALDPVPQDASTK	1013,494	446,170
Q9C0C7	Activating molecule in BECN1-regulated autophagy protein 1 (**AMBRA1**)	SLGGPLSSHPSR	597,815	315,166
		**LMAEGGMTAVVQR**	**681,847**	**862,380**
